# Research without prior consent procedure and intervention effect on mortality in critical care: a meta-epidemiological study of randomized controlled trials

**DOI:** 10.1186/s13054-025-05480-x

**Published:** 2025-07-24

**Authors:** Geoffroy Hariri, Jacqueline Louie, Aqsa Khan, Peggy Tahir, Guillaume L. Martin, Agnès Dechartres, Matthieu Legrand

**Affiliations:** 1 Department of Anesthesia and Perioperative Care, Division of Critical Care Medicine, University of California, San Francisco, 521 Parnassus Ave, San Francisco, CA 94143 USA; 2 Sorbonne Université, GRC 29, Groupe de Recherche Clinique en Anesthésie Réanimation médecine Périopératoire, ARPE, Paris, F-75012 France; 3 Sorbonne Université, INSERM, Institut Pierre Louis d’Épidémiologie et de Santé Publique, AP-HP, Sorbonne Université, Hôpital Pitié-Salpêtrière, Département de Santé Publique, Paris, France; 4 UCSF Library, University of California, San Francisco, CA USA; 5 French Clinical Research Infrastructure Network Initiative- Cardio Renal Clinical Trialists Network, Nancy, France

**Keywords:** Research without prior consent, Critical care, Meta-epidemiological study, Randomized controlled trials, Ethics

## Abstract

**Background:**

In critical care randomized controlled trials (RCTs), obtaining informed consent from patients or proxies can be challenging and may delay randomization, potentially affecting intervention efficacy. Research without prior consent (RWPC) procedures are increasingly used to facilitate timely inclusion but their impact on trial outcomes remains uncertain. We aimed to assess whether RWPC procedures are associated with differences in intervention effects on mortality in critical care RCTs.

**Methods:**

We searched PubMed and the Cochrane Database of Systematic Reviews from inception to August 1, 2024. We included meta-analyses of RCTs evaluating therapeutic interventions in critically ill adults, reporting mortality as a primary or secondary outcome. We conducted a meta-epidemiological study using a two-step approach. First, we calculated the ratio of odds ratios (ROR) within each meta-analysis to compare the effect of interventions on mortality between RCTs using RWPC and those using standard consent. Second, we pooled these RORs across meta-analyses using a random-effects model. Secondary outcomes included the delay from eligibility to randomization and the recruitment rate.

**Results:**

We included 42 meta-analyses comprising 323 RCTs and 103,011 patients, of which 59 RCTs (18%) used a RWPC procedure. Trials using RWPC were more recent (median year: 2015 [2008–2019] vs. 2012 [2007–2017]; *p* < 0.01), larger (sample size: 203 [101–605] vs. 72 [40–162]; *p* < 0.01), more frequently multicenter (80% vs. 43%; *p* < 0.01), and had lower overall risk of bias. There was no significant difference in intervention effect on mortality between trials with and without RWPC (pooled ROR, 1.05 [95% CI 0.83–1.34]; I²=71.7%). RWPC was associated with shorter time to randomization (3 [1−9] vs. 11 [4−23] hours; *p* < 0.01) and higher recruitment rates (9.6 [4.7–18.7] vs. 4.5 [1.9–8.6] patients/month; *p* = 0.01).

**Conclusions:**

In critical care RCTs, RWPC procedures were not associated with differences in intervention effect on mortality but were linked to shorter time to randomization and higher recruitment rates.

**Supplementary Informations:**

The online version contains supplementary material available at 10.1186/s13054-025-05480-x.

## Introduction

In critical care research, designing trials that reflect real-world clinical conditions is particularly challenging. Critically ill patients often experience rapid clinical deterioration or improvement, making the timing of interventions crucial. In such settings, delays between eligibility assessment and randomization—particularly those introduced by the need to obtain informed consent—can interfere with the delivery of time-sensitive treatments and affect trial outcomes. Such discrepancy may have an impact on trial results and yield differences with real-world applications [1]. This may be one of the reasons that could explain why among the many RCTs performed in critical care, only a small proportion yielded positive results, raising questions about their research methods [2]. Several explanations for the lack of positive results have been proposed, including too optimistic effect sizes when calculating sample size. Another challenge when conducting RCTs in critical care is the time between eligibility and randomization. Life-threatening conditions exhibit rapid progression, with swift improvement or deterioration. In this context, obtaining informed consent, especially in patients unable to consent for themselves due to critical illness can introduce a significant delay between eligibility assessment and randomization. This may lead to missing the optimal therapeutic window of the evaluated intervention [3−8].

Research without prior consent (RWPC) procedure allows for the randomization of patients without obtaining prior consent. The 2024 revised World Medical Association Declaration of Helsinki [9] permits this procedure in patients unable to give their free and informed consent if the research cannot be delayed to improve the quality of research in an emergency setting. This approach may reduce the time between eligibility and randomization and increase the chances of an intervention impacting outcomes. However, the potential impact of RWPC procedures on intervention effects remains unexplored.

In this study, we aimed to assess the association between the RWPC procedure and intervention effect on mortality, and its influence on time to randomization and recruitment rate as secondary outcomes in RCTs involving critically ill patients. We hypothesized that trials using RWPC procedures would show larger intervention effects on mortality, due to earlier randomization allowing better alignment with the therapeutic window in critical setting.

## Methods

### Study design

We used a meta-epidemiological approach, the reference method to identify bias in RCTs [10]. This study was conducted and reported in accordance with methodological guidance for meta-epidemiological research, as recommended by Murad and Wang [11]. This method consists in assessing whether a given characteristic is associated with intervention effect in a sample of meta-analyses. First, we performed a systematic review to identify all meta-analyses of RCTs assessing a therapeutic intervention and reporting mortality as an outcome in patients with clinically unstable conditions in critical care settings. Then, we assessed the procedure of consent reported in each included RCT in particular whether a RWPC procedure was implemented or not. Finally, we compared the intervention effect between RCTs with and without an RWPC procedure using a meta-epidemiological analysis.

## Search strategy

We identified systematic reviews with meta-analysis evaluating therapeutic interventions on mortality in patients with clinically unstable conditions ultimately managed in ICU, although inclusion in some trials could occur in highly monitored settings prior to ICU admission, by searching Medline via PubMed and the Cochrane Database of Systematic Reviews using dedicated search terms (Supplementary Material 1: Supplemental 1) from inception to August 1 st, 2024. As this is a meta-epidemiological study, which aims to assess methodological patterns rather than to comprehensively identify all meta-analyses on a specific clinical topic, a focused search strategy using two major databases was considered sufficient. This process was validated by a senior researcher having previously published a number of meta-epidemiological studies [12−14]. We focused on critical care conditions for which the timing of intervention may affect the outcomes, namely shock (sepsis, cardiogenic or hemorrhagic), sepsis, acute respiratory distress syndrome (ARDS), out-of-hospital cardiac arrest (OHCA), and trauma.

## Study selection

### Selection of meta-analyses

We included meta-analyses of RCTs involving critically ill adult patients assessing a therapeutic intervention and evaluating mortality as a primary or secondary outcome. Meta-analyses of unpublished data, involving individual-patient data, and those of cluster or crossover RCTs have been excluded. Meta-analyses involving fewer than 3 RCTs have also been excluded, as a minimum of three RCTs is required to conduct a meta-epidemiological analysis. Overlapping meta-analyses, defined as those sharing 3 RCTs or more, were identified and we included the meta-analysis with the highest number of RCTs.

Two reviewers (GH and PT) independently assessed the eligibility of retrieved references after removing duplicates. Discrepancies were resolved by discussion with a third reviewer (ML) to reach consensus.

## Selection of RCTs

All individual RCTs included in the meta-analysis of mortality were searched and included except those involving participants younger than 18 years old.

Eligibility criteria applied at the meta-analysis and RCT levels and corresponding justifications are presented in Supplementary Material 1: S. Table 1.

**Table 1:** Main characteristics of the included meta-analyses (*n* = 42)

Characteristics	*N* = 42
Source, n (%)	
- Cochrane reviews	3 (7)
- Non-Cochrane reviews	39 (93)
Year of publication, med [Q1-Q3]	2020 [2017–2023]
Funding, n (%)	
- None	19 (45)
- Public	16 (38)
- Private	3 (7)
- Mixed	2 (4)
- Not reported	2 (4)
Medical condition, n (%)	
- Sepsis/Septic shock	23 (54)
- ARDS	9 (21)
- Cardiac arrest	4 (9)
- Cardiogenic shock	4 (9)
- Trauma	2 (4)
Intervention type, n (%)	
- Pharmacological	17 (40)
- Non-pharmacological	25 (60)
Control group, n (%)	
- Placebo	14 (33)
- Usual care	4 (9)
- No intervention	2 (4)
- Other intervention	22 (53)
Primary outcome, n (%)	
- Mortality	40 (95)
- Other	2 (5)
Time points of mortality assessment, n (%)	
- In ICU	1 (2)
- 28/31 d	17 (40)
- In hospital	3 (7)
- Overall	18 (43)
- Other	3 (7)
Evaluation of the risk of bias, n (%)	
- RoB 1	20 (48)
- RoB 2	14 (33)
- Jadad scale	6 (27)
- Not reported	2 (2)
Number of trials in the meta-analysis of mortality, med [Q1-Q3]	6 [4–12]

ARDS: Acute respiratory distress syndrome. ICU: Intensive care unit. RoB: Risk of bias

### Assessment and definition of RWPC

For each RCT, we collected from whom consent was obtained (e.g., patient, surrogate decision maker or either), whether consent was obtained orally or in writing, and whether a RWPC procedure was used. RWPC was defined as the inclusion of a patient in the trial before obtaining his/her consent or the consent of a surrogate decision maker (SDM). It also included trials where consent was formally waived with ethical approval (i.e “omitted consent”), meaning that no consent was obtained at all during the trial, in compliance with an ethical approvement. These trials were categorized as using RWPC [15]. If no details on how the consent was reached were provided in the trial, we contacted the authors. Without a response from the authors, we excluded the RCT from all analyses.

## Data extraction

Two reviewers (GH and JL) independently extracted the data; any disagreements were resolved by discussion with a third reviewer (ML). Two data collection forms have been developed: one for meta-analyses and one for individual trials (Supplementary material 2). Data for RCTs have been directly extracted from the RCT reports. When the full text was unavailable, the authors were contacted. In case of no response, we excluded the RCT of the analysis.

For each meta-analysis, the following data were extracted: date of publication, journal, funding sources, number of trials included in the meta-analysis of mortality, medical condition, name and type of intervention (pharmacological or non-pharmacological) assessed in each groups, whether mortality was a primary outcome and time point of mortality assessment: in ICU, 28–31 days, 90 days, in hospital or overall, tool used to assess risk of bias, results of the meta-analysis for mortality (combined estimate with confidence intervals (CIs) and heterogeneity assessed with the I^2^ and Cochran Q chi square test).

For each RCT included in the meta-analysis of mortality, we collected: date and journal of publication, including whether it was among the five highest-ranking journals in general medicine (i.e. The New England Journal of Medicine, Journal of the American Medical Association, The Lancet, Nature Medicine, The British Medical Journal), funding source, country, sample size, number of centers, main inclusion criteria, details of the consent process: written or oral, whether it was acquired directly from the patient or from a SDM, if the consent was obtained once the patient’s consciousness was regained, from a SDM or if it was omitted in case of RWPC procedure, time from eligibility to randomization, recruitment rate, defined as the ratio of the total study duration to the sample size, number of consent withdrawals, whether estimated consent withdrawals were anticipated in the sample size calculation, whether mortality was a primary outcome and time point of mortality assessment: in ICU, 28–31 days, 90 days, in-hospital or overall, results for mortality with the number of events and analyzed participants in the experimental and control groups. The corresponding authors were contacted in case of missing information about the consent procedure and the time interval from eligibility to randomization. The risk of bias of each trial was extracted directly from the meta-analysis when the Cochrane risk of bias tools 1.0 or 2.0 were used. If another tool was used or if the risk of was not reported, two reviewers (GH/ML) independently assessed the risk of bias with the RoB 2.0 from the RCT report.

## Data synthesis

### Estimation of intervention effects within meta-analyses

We estimated the intervention effects within each meta-analysis as odds ratios (OR) and 95% confidence intervals (CIs). Mortality was recorded so that an OR < 1 indicated a beneficial effect of the experimental intervention. We used random-effects models to combine intervention effects across RCTs within each meta-analysis [16]. RCTs with no events or all events in both groups did not contribute to the analysis. Heterogeneity across RCTs was assessed with the Cochran Q chi-square test, I^2^ statistic, and between-study variance τ^2^.

### Publication bias assessment

We used contour-enhanced funnel plots to assess the presence of small-study effects in each meta-analysis including 10 or more randomized controlled trials. This approach helps distinguish asymmetry due to publication bias from that due to other causes by highlighting statistical significance contours [17].

### Meta-epidemiological analysis

We used the two-step approach described by Sterne et al. [18]. First, we calculated, within each meta-analysis, the ratio of ORs (ROR) — the ratio of the intervention-effect OR in RCTs using RWPC procedure to the OR in RCTs not using RWPC procedure by using a random-effects meta-regression model to incorporate between-trial heterogeneity. An ROR < 1 indicates larger intervention effect estimates for trials using a RWPC procedure. Then, we estimated the combined ROR across meta-analyses and its 95% CI by using a random effects meta-analysis model. Heterogeneity across RORs was quantified with the Cochran Q chi-square test, I^2^ statistic, and between–meta-analysis variance τ^2^.

### Subgroup and sensitivity analyses

We performed subgroup analyses according to the type of intervention (pharmacological versus non-pharmacological) and the medical condition (OHCA, ARDS, Sepsis, Trauma, Cardiogenic shock). We performed an interaction test to assess whether the ROR varied between subgroups. To control for potential confounders, sensitivity analyses were performed by adjusting the meta-regression models for sample size, the overall risk of bias (high or unclear risk/some concerns vs. low risk), the single-center vs. multicenter designs and a publication date before versus after 2010.

Because our study is meta-epidemiological in nature and does not estimate clinical treatment effects, we did not apply the GRADE approach, which is primarily intended for intervention studies. Instead, we assessed the reliability and consistency of our findings through the examination of heterogeneity and through prespecified sensitivity analyses. Data were analyzed using R version 4.4.2 [19]. A two-sided p-value less than 0.05 was considered statistically significant.

## Results

### Selection and general characteristics

**Fig. 1:**
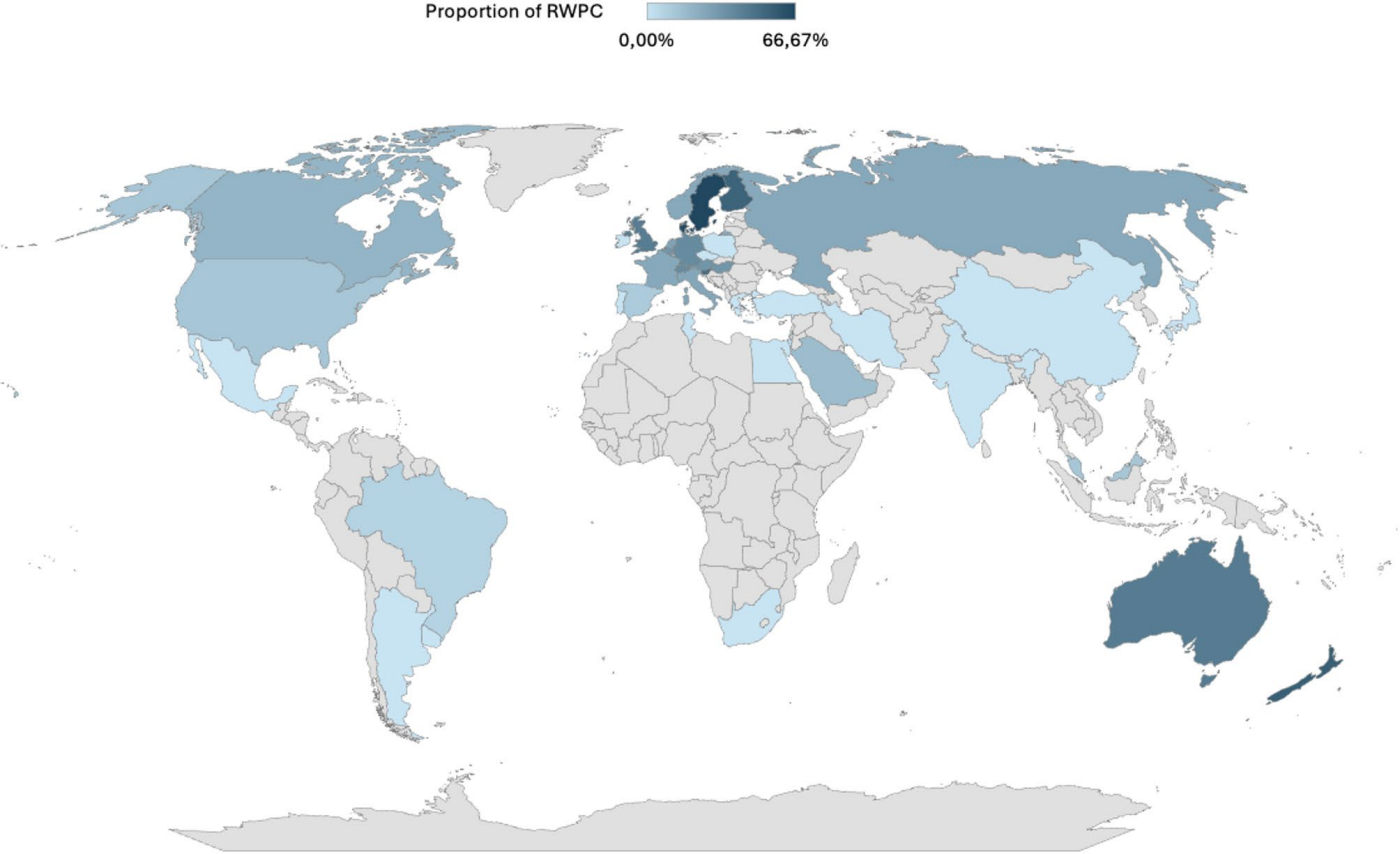
Rate of trials using RWPC procedure in each country

Of the 1910 references identified by the electronic search, 42 meta-analyses were selected comprising 356 RCTs (Table 1, Supplementary Material 1: S. Figure 1 and S.Table 2). After contacting authors for missing consent details, 25 RCTs were excluded for insufficient information, along with 3 duplicates, 1 pediatric trial, 1 retracted trial, and 3 inaccessible trials. Importantly, the exclusion of the single pediatric trial did not affect the selection of meta-analyses based on the highest number of RCTs, as no alternative meta-analysis with a greater number of eligible adults RCTs existed. Finally, 323 RCTs were included in the analysis involving 103,011 patients. The main medical condition was sepsis in 223 (69%) trials, followed by ARDS in 58 (18%) trials. Most of the interventions evaluated were pharmacological (*n* = 210, 65%), and mortality was the primary outcome in 115 (35%) trials.

**Table 2:** Main characteristics of included RCTs according to the use of research without prior consent (RWPC) or not

Characteristics	RWPC *N* = 59	Non RWPC *N* = 264	*P*
Top 5 journal, n (%)	26 (45)	44 (17)	< 0.01
Year of publication, med [Q1-Q3]	2015 [2008–2019]	2012 [2007–2017]	0.03
Funding, n (%)			< 0.01
- None	0 (0)	21 (8)	
- Public	38 (63)	87 (34)	
- Private	10 (16)	33 (13)	
- Mixed	4 (8)	17 (6)	
- Not reported	7 (11)	56 (22)	
Number of centers, n (%)			< 0.01
- Single-center	12 (20)	151 (57)	
- Multicenter	47 (80)	113 (43)	
Sample size, med [Q1-Q3]	203 [101–605]	72 [40–162]	< 0.01
Medical condition, n (%)			< 0.01
- Cardiac arrest	19 (32)	2 (1)	
- ARDS	8 (13)	50 (19)	
- Sepsis/Septic shock	27 (47)	196 (73)	
- Cardiogenic shock	3 (4)	10 (5)	
- Trauma	2 (3)	6 (2)	
Intervention type, n (%)			< 0.01
- Pharmacological	17 (28)	193 (74)	
- Non-pharmacological	42 (71)	70 (27)	
Control group, n (%)			< 0.01
- Placebo	9 (15)	101 (38)	
- Standard of care	1 (1.7)	45 (17)	
- No intervention	8 (14)	2 (0.8)	
- Other intervention	41 (69)	116 (44)	
Primary outcome, n (%)			< 0.01
- Mortality	32 (53)	83 (32)	
- Other	27 (46)	181 (68)	
Primary outcome positive, n (%)	22 (37)	114 (44)	0.63
Mortality outcome positive, n (%)	6/32 (18)	16/83 (19)	0.47
Consent withdrawn (%), med [Q1-Q3]	1.3 [0.2–2.3]	0 [0–0.2]	0.049
Overall Risk of Bias, n (%)			< 0.01
- Low	30 (51)	74 (28)	
- High/Unclear or Some concerns	29 (49)	190 (72)	

ARDS: Acute respiratory distress syndrome

### Consent procedures in RCTs

**Fig. 2:**
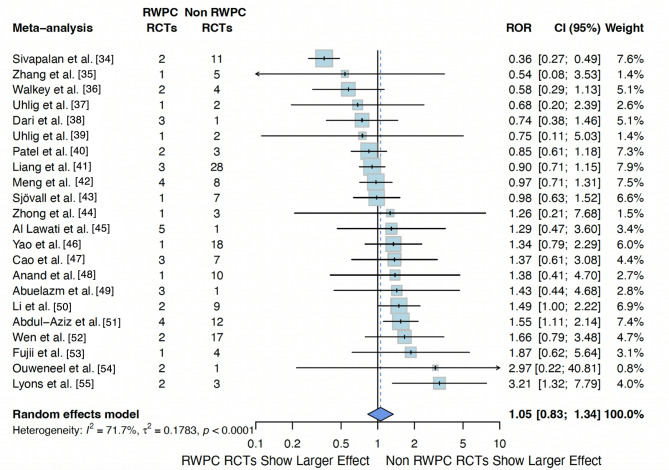
Difference in intervention effect estimate on mortality in 22 meta-analyses including 47 RCTs using a RWPC procedure and 157 not using a RWPC procedure

Among all RCTs, 59 (18%) used a RWPC procedure and 264 (82%) did not. During the screening and data extraction phases, five disagreements occurred between the two reviewers (GH and PT). Four of these concerned the classification of studies as involving research without prior consent (RWPC) or not. Following discussion with a third reviewer (ML), all four were ultimately classified as RWPC, as ethical approval had been granted to include patients under this framework. The fifth disagreement concerned the timing of consent in a trial initially considered as non-RWPC. Upon review (with ML), this study was excluded, as consent had been obtained before the participant met eligibility criteria. All disagreements were resolved through discussion and consensus. Details of the consent source are provided in Supplementary Material 1: S.Table 3. The proportion of trials using a RWPC procedure varies by country (Fig. 1) and has increased since 1980 (Supplementary Material 1: S. Figure 2).

### Comparison of characteristics between RCTs with and without RWPC procedure

Characteristics of trials according to the use of a RWPC procedure are detailed in Table 2. Trials using a RWPC were more likely to be published recently (median year of publication 2015 [2008–2019] vs. 2012 [2007–2017]; *p* < 0.01), to have a larger sample size (median 203 [101–605] vs. 72 [40–162]; *p* < 0.01), to be multicenter (*n* = 47, 80% vs. *n* = 113, 43%; *p* < 0.01). The rate of participants withdrawing their consent was higher when a RWPC procedure was used (1.3 [0.2–2.3] vs. 0 [0–0.2] %; *p* = 0.049). Anticipated consent withdrawals were integrated in the sample size calculation in 8 (13%) trials with RWPC procedure and in 3 trials (0.1%) without. Trials with RWPC also had a lower overall risk of bias (Low RoB: *n* = 30, 51% vs. *n* = 74, 28%; *p* < 0.01) (Table 2).

### Comparison of intervention effect between RCTs with and without RWPC procedures

#### Primary analysis

Twenty-two meta-analyses (204 RCTs) with at least one RCT with RWPC and one RCT without RWPC, were included in the primary analysis. We found no significative difference in intervention effect on mortality between trials with a RWPC procedure versus those without (ROR: 1.05, 95% CI 0.83–1.34) with high heterogeneity (I^2^: 71.7%; P 0.01; between–meta-analysis variance t^2^ = 0.1783). (Fig.2).

### Publication bias assessment

**Fig. 3:**
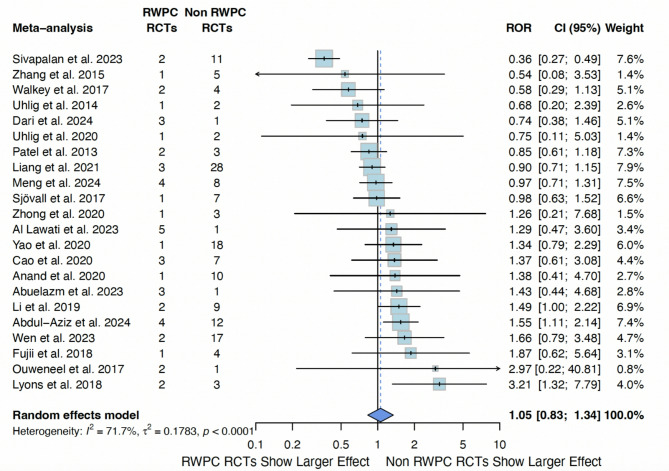
Time interval between eligibility and randomization according to the use of a RWPC procedure in different medical conditions

Of the 22 meta-analyses included in the meta-epidemiological study, nine contained more than 10 randomized controlled trials, allowing for an assessment of small study effects. Evidence of asymmetry consistent with a publication bias was observed in six of these meta-analyses, as demonstrated by contour-enhanced funnel plots (Supplementary Material 1: S.Figures 3 -11).

### Subgroup and sensitivity analyses

The subgroup analysis by type of intervention revealed a combined ROR of 0.88 (95% CI 0.61–1.28) across the 12 meta-analyses assessing non-pharmacological interventions and 1.24 (95% CI 0.97–1.59) across the 10 meta-analyses evaluating pharmacological interventions. The interaction test was not statistically significant (*p* = 0.14)(Supplementary Material 1: S.Figure 12). . Similarly, the subgroup analysis by clinical condition showed consistent results. In patients with OHCA, the combined ROR was 1.35 [95% CI 0.62–2.93], with no significant heterogeneity (I² = 0%, *p* = 0.90). In the ARDS subgroup, the combined ROR was 0.88 [95% CI 0.57–1.36], also without heterogeneity (I² = 0%, *p* = 0.47). Among patients with sepsis, the combined ROR was 1.00 [95% CI 0.74–1.34], with substantial heterogeneity (I² = 62%, *p* < 0.001). The trauma subgroup included only a single study, which precluded meta-analytic pooling. In cardiogenic shock, the combined ROR was 1.21 [95% CI 0.26–5.62], with no observed heterogeneity (I² = 0%, *p* = 0.40). There was no significant interaction between subgroups (p for interaction = 0.12) (Supplementary Material 1: S. Figure 13). Sensitivity analyses were consistent with the primary analysis (Supplementary Material 1: S.Figure 14).

#### Time interval between eligibility and randomization

The time from patient eligibility to randomization was reported in 133 trials (41%). Overall, the RWPC procedure was associated with a significantly shorter time interval between eligibility and randomization (3 [1–9] vs. 11 [4–23] hours; *p* < 0.01). This result was consistently found across all medical conditions (Fig. 3).

### Recruitment rate

The recruitment rate was reported in 258 trials (80%). The use of an RWPC procedure significantly increased the overall patient recruitment rate (9.6 [4.7–18.7] vs. 4.5 [1.9–8.6] patients per month; *p* = 0.01), but not by center (0.8 [0.4–1.5] vs. 1.4 [0.6–3.8] patients per month per center; *p* = 0.09). In a stratified analysis, the use of RWPC was associated with a substantial and statistically significant increase in recruitment rate in multicenter trials (+ 16.6 patients per month, *p* = 0.010), whereas no such effect was observed in single-center trials (*p* = 0.97). To explore potential explanations for this difference, we compared trial characteristics between single-center and multicenter trials. Compared to single-center trials, multicenter trials more frequently included patients with ARDS or cardiac arrest and assessed non-pharmacological interventions (Supplementary Material 1: S. Table 4).

## Discussion

In this meta-epidemiological study including 42 meta-analyses and 323 RCTs assessing mortality in 103,011 critically ill patients, nearly one-fifth of trials reported a RWPC procedure. Our main finding was the absence of a significant difference in intervention effect on mortality between RWPC and non-RWPC trials. Similarly, no significant differences were observed in the subgroup analyses based on the type of intervention or the medical condition. RWPC trials were more recent, had larger sample sizes, were more frequently multicenter, and had a lower overall risk of bias. RWPC procedures were associated with shorter time from eligibility to randomization and a higher recruitment rate.

Among the 356 RCTs initially assessed, 25 (7%) were excluded due to a lack of information regarding the consent process. These findings highlight that in critical care RCTs, reporting of consent and from whom it was obtained (patient, SDM, or either) is consistently reported. However, details on how consent was obtained (e.g., written, oral) are much less frequently provided. This gap in reporting limits the transparency and reproducibility of research.

Overall, less than one of five of included trials use a RWPC procedure. The RWPC procedure was developed to address the practical challenges of conducting clinical research in emergency and critical care settings. The concept of enrolling a patient in research without his prior consent was introduced in 1991 by Levine [20]. This approach is now endorsed by ethical and regulatory frameworks such as the Declaration of Helsinki [9], CIOMS guidelines [21], and national regulations in several countries [22]. RWPC is intended to preserve the ethical integrity of emergency research by combining timely intervention with post-enrollment consent procedures. However, the use of RWPC raises several ethical challenges, as in interventional trials it involves not a “deferred” consent for the intervention itself, but rather for the use of the collected data [23].

We observed no significant difference in intervention effect on mortality between RWPC and non-RWPC trials. Several factors may explain our results. First, the low rate of positive meta-analyses in our study is noteworthy. Among the meta-analyses included in our meta-epidemiological study, only two showed a statistically significant result in favor of the experimental intervention, which is consistent with the persistent lack of effective interventions to reduce mortality in critical care over the past decades [2]. A RWPC procedure is unlikely to influence the intervention’s effectiveness if the intervention itself is not effective. Second, we observed that non-RWPC trials tended to be smaller, more frequently single-center, and older compared to RWPC trials. These characteristics have been associated with larger intervention effect estimates in RCTs [12, 24, 25]. Another notable difference was the higher overall risk of bias in trials without RWPC procedure. Meta-epidemiological studies have shown that a higher risk of bias is also associated with exaggerated intervention effects in RCTs [26, 27]. Finally, post hoc analysis of three multicenter trials including 1981 patients, found that those unable to consent had higher severity of illness and mortality [28]. Altogether, these characteristics may offset the RWPC procedure’s potential positive impact on intervention effect, resulting in no significant difference in intervention effects between RWPC and non-RWPC trials.

Heterogeneity across the included meta-analyses was substantial, potentially impacting the pooled estimates and limiting the precision of the results. This variability likely reflects differences in medical conditions and types of interventions, as heterogeneity was reduced within each analyzed subgroup.

Another observation in our study is the association between the use of RWPC and a shorter time from eligibility to randomization and a higher recruitment rate in multicentric trials. This finding, although based on a limited subset of trials, may reflect more streamlined recruitment processes in emergency contexts. A shorter time from recognition to intervention has been associated with reduced mortality in patients with sepsis or septic shock [1, 29]. The improvement of recruitment rate align with the report by Annane et al., which showed that the implementation of an RWPC procedure in an RCT involving septic shock patients, resulted in an increased recruitment rate from 4 to 10 patients per month [30]. However, this should not be interpreted as a recommendation to systematically waive prior consent in trials in critical care setting. Consent for research can and should be designed to reflect the realities of clinical care, including approaches that align with how consent is obtained for treatment decisions in critical care. Our findings underscore the need for further empirical and ethical research on optimizing consent processes in time-sensitive settings.

Interestingly, the association between RWPC use and higher recruitment rate was observed only in multicenter trials. One possible explanation is that, in multicenter settings, logistical variability and delays related to consent procedures may be more pronounced due to differences in local protocols, staff training, and administrative processes. RWPC may help standardize and streamline enrollment procedures across sites, thereby enhancing overall recruitment efficiency. In contrast, single-center trials are often conducted by tightly coordinated teams within a unified institutional framework, where consent processes are more easily integrated into the clinical workflow. As such, the operational benefits of RWPC may be less impactful in single-center settings. Another hypothesis to explain the association between RWPC and recruitment rate observed only in multicenter trials is the nature of the interventions and medical conditions more commonly studied in these settings. Multicenter trials more frequently included patients with ARDS or cardiac arrest and assessed non-pharmacological interventions, where obtaining prior consent is particularly challenging due to the urgency and severity of the clinical context.

Consent for data collection and use should be obtained from the patient and/or their SDM, whether the RWPC procedure is used or not. In the context of the RWPC procedure, once the SDM is present and/or the patient regains the ability to consent, follow-up consent is sought, and the patient or his/her SDM is free to exit the trial. Our results showed that RWPC is associated with a modest but significantly higher rate of consent withdrawal (1.3%). This finding highlights the importance of accounting for this factor when calculating sample size for RCTs. Although the RWPC procedure appears generally well-accepted by ICU patients [31− 33], a study by van der Wal et al. exploring thoughts of former ICU patients having participated in an RCT with a RWPC procedure found that 59% of them were unaware of their participation [33]. This underscores the importance of enhancing post-study communication and community engagement to improve patient awareness and understanding.

Our findings suggest that RWPC is an interesting approach in critical care trials involving patients with unstable conditions. The association between RWPC and a shorter time between eligibility and randomization and faster recruitment may enhance the feasibility of conducting trials in time-sensitive settings.

However, these potential operational benefits did not translate into improved patient outcomes in our analysis, and our findings do not support the hypothesis that RWPC use is associated with reduced mortality. It is possible that differences in patient severity influenced these results, but severity was insufficiently and inconsistently reported across trials to allow meaningful adjustment or subgroup analysis. Therefore, RWPC use must be carefully justified based on the clinical context, the anticipated urgency of the intervention, and the inability to obtain informed consent in a timely manner. While the overall rate of consent withdrawal was low, the fact that a substantial proportion of patients were unaware of their participation underscores the importance of improving post-enrollment communication and transparency.

Future research should aim to identify the specific clinical situations and intervention types where RWPC most closely aligns with care and provides the greatest added value. Understanding where and when RWPC enhances both trial feasibility and ethical acceptability will help refine its application and guide ethical oversight. Moreover, studies exploring patient, family, and public perspectives on RWPC are essential to inform best practices for follow-up consent and optimize trust in research conducted in acute care settings. Moving forward, future trials should systematically report from whom, by whom and how the consent was obtained. Such reporting would enhance the methodological and ethical rigor of clinical trials.

Our study has several limitations, which we present below at both the study and meta-epidemiological levels.

At the study level, first only 41% of the included RCTs reported the time from eligibility to randomization, which limits the strength and interpretability of analyses related to this outcome. Although we attempted to mitigate this limitation by contacting corresponding authors to obtain missing data, the implications of our findings regarding time to randomization remain uncertain, particularly for pragmatic trials, which may adopt consent procedures more closely aligned with routine clinical practice. Second, some trials reported mortality as a secondary outcome, even though the intervention was not expected to influence it, potentially leading to dilution of effect estimates and reduced statistical power. Third, we excluded trials involving pediatric patients to maintain population homogeneity, which may limit the generalizability of our findings to broader patient populations.

At the meta-epidemiological level, our search strategy, although comprehensive, may have missed some relevant meta-analyses. However, we believe this is unlikely to have introduced systematic bias. In addition, our focus on mortality aimed to ensure a homogeneous and consistently reported outome, but it may have overlooked other clinically important outcomes. We chose mortality as the primary outcome because it is the most consistently reported, objective outcome across trials involving unstable patients, allowing for valid comparisons in a meta-epidemiological framework. Outcomes such as organ failure scores, while clinically relevant, are more variable across settings and subject to differing practices, which could have introduced additional heterogeneity and bias into the analysis. We also excluded RCTs with insufficient information on the consent process despite multiple follow-up attempts. This may have limited the comprehensiveness of our dataset, but it was necessary to ensure accurate classification of RWPC procedures. Finally, due to the nature of meta-epidemiological studies, subgroup analyses could only be performed at the meta-analysis level and not based on individual trial characteristics such as geographic region or disease severity.

## Conclusion

We found no significant difference in intervention effects on mortality between trials with a RWPC procedure and those without. While RWPC was associated with a shorter time between eligibility and randomization and with faster recruitment in a subset of trials, this operational advantage must be weighed against the ethical importance of informed consent prior to randomization. These findings do not imply that RWPC should systematically replace conventional consent procedures. Rather, they highlight the need for continued reflection on how best to balance ethical requirements and feasibility in time-sensitive clinical research settings.

## Supplementary Informations

Supplementary Material 1

Supplementary Material 2

Supplementary Material 3

## Data Availability

Data available: Yes Data types: Data (not involving human participants) How to access data: geoffroy.hariri@aphp.fr. When available: With publication.

## Abbreviations

ARDS: Acute respiratory distress syndrome
Cis: Confidence intervals
ICU: Intensive care unit
OHCA: Out-of-hospital cardiac arrest
OR: Odds ratios
RCT: Randomized controlled trials
ROR: Ratio of odds ratios
RoB: Risk of bias
RWPC: Research without prior consent
SDM: Surrogate decision maker

## **Declaration**

### Patient and public involvement statement

Patients and the public were not involved in the design, conduct, analysis, or dissemination of this study.
