# Supplementary material for: Research without prior consent procedure and intervention effect on mortality in critical care: a meta-epidemiological study of randomized controlled trials
Source: Crit Care. 2025 Jul 24;29:323. doi: 10.1186/s13054-025-05480-x (PMC12291516; doi:10.1186/s13054-025-05480-x)
Supplement: Supplementary file 1 — Supplementary Material 1 [file 13054_2025_5480_MOESM1_ESM.docx]

**Additional file**

**Research without prior consent and intervention effect on mortality in critical care: A meta-epidemiological study of randomized controlled trials**

Geoffroy Hariri, MD, PhD^1^ ; Jacqueline Louie, BA; Aqsa Khan, MBBS; Peggy Tahir MLIS,MA; Guillaume L Martin, MD ; Agnès Dechartres, MD, Ph^3^ ;

Matthieu Legrand, MD, PhD^1^

**Supplemental 1.** Search terms

1. “Systematic review” [tiab]
2. “Systematic reviews” [tiab]
3. “Systematic review” [pt]
4. “meta-analysis” [tiab]
5. “meta analysis” [pt]
6. #1 OR #2 OR #3 OR #4 OR #5
7. “shock” [tiab]
8. “Acute respiratory distress syndrome” [tiab]
9. “ARDS” [tiab]
10. “Cardiac arrest” [tiab]
11. “trauma” [tiab]
12. “Sepsis” [tiab]
13. “acute respiratory distress syndrome” [MeSH Terms]
14. “ards, human”[MeSH Terms]
15. “sepsis”[MeSH Terms]
16. “cardiac arrest”[MeSH Terms]
17. “septic shock”[MeSH Terms]
18. “cardiogenic shock”[MeSH Terms]
19. “hemorrhagic shock”[MeSH Terms]
20. #7 OR #8 OR #9 OR #10 OR #11 OR #12 OR #13 OR #14 OR #15 OR #16 OR #17 OR #18
21. “Intensive care” [tiab]
22. “ICU” [tiab]
23. “Critical care” [tiab]
24. “Critical care” [mh]
25. “Critically ill” [tiab]
26. #21 OR #22 OR #23 OR #24 OR #25
27. #6 AND #21 AND #26
28. “child*” [tiab]
29. “infant” [tiab]
30. “Neonat*” [tiab]
31. #28 OR #29 OR #30
32. #27 NOT #31

|  | Eligibility criteria | Justification |
| --- | --- | --- |
| Meta-analysis level inclusion criteria | Meta-analyses of RCTs | To ensure high internal validity and comparability across included studies |
|  | Adult patients with a clinically unstable condition (all types of shock (septic, cardiogenic or hemorrhagic), ARDS, OHCA, sepsis and trauma) | To ensure high internal validity and comparability across included studies |
|  | Evaluates a therapeutic intervention | To restrict the analysis to interventions with potential clinical impact |
|  | Mortality as a primary or secondary outcome | To ensure consistency and objectivity in outcome assessment |
| Meta-analysis level exclusion criteria | Excludes unpublished data, individual patient data, and non-standard designs (cluster or crossover RCTs) | To ensure methodological consistency and avoid bias introduced by different trial designs |
|  | Fewer than 3 RCTs included | To allow meaningful meta-epidemiological comparisons and estimation of between-study heterogeneity |
| RCT level inclusion criteria | Includes in selected meta-analyses | To maintain consistency with the meta-analysis dataset and allow for accurate extraction of trial characteristics |
| RCT level exclusion criteria | Involving participants <18 years | To ensure population homogeneity and relevance to adult critical care |
|  | No information of consent procedure | To ensure availability of key methodological data necessary for the study's primary objective |
|  | Duplicate RCTs | To avoid data duplication and maintain accuracy in the dataset |
|  | Retracted RCTs/ No full text available | To enable full data extraction and quality |

**S. Table 1:** Eligibility criteria applied at the meta-analysis and RCT levels, with corresponding justifications.

RCT: Randomized controlled trial ; ARDS: Acute respiratory distress syndrome. OHCA: Out of Hospital Cardiac Arrest.

**S.Figure 1.** Flow chart of the selection of meta-analyses and the RCTs included

Records identified from

MEDLINE (n = 1863)

Cochrane Database of Systematic Review (n = 49)

Records excluded (n = 1695)

RCT excluded:

No information on consent (n = 25)

No full text (n = 3)

Retracted (n=1)

Pediatric population (n = 1)

Duplicates (n = 3)

Reports excluded:

Overlap MA (n = 112)

No MA (n = 7)

No clinically unstable condition (n = 32)

MA including less than 3 studies (n = 10)

Mortality outcome not available (n = 6)

No RCT included (n = 8)

**Included**

**323 unique RCTs**

**Meta-analyses included**

**(n = 42)**

**(n = 356 RCTs)**

**Screening**

Records screened

(n = 1912)

Reports assessed for eligibility

(n = 217)

**Identification**

MA : Meta-analysis. RCT : Randomized controlled trial

**S.Table 2.** Detailed characteristics of included meta-analyses

| First author.  Journal | Year | Intervention | Control treatment | Time point of mortality assessment | RoB assessment | Number of trials | MA result | Heterogeneity  I2 (%) |
| --- | --- | --- | --- | --- | --- | --- | --- | --- |
| ARDS | | | | | | | | |
| Anand et al.  Thorax [1] | 2020 | Mucoactive agent | Standard of Care | Overall | RoB 1.0 | 12 | OR : 0.75  [0.52; 1.08] | 13 |
| Cao et al.  Emergency Medical International [2] | 2020 | Prone position | Supine position | Overall | Jadad Scale | 11 | RR : 0.87  [0.75; 1.00] | 40,5 |
| Feng et al.  Medicine [3] | 2022 | Dexamethasone | Standard of Care | Overall | RoB 1.0 | 3 | OR : 0.62  [0.44; 0.88] | 0,3 |
| Luo et al.  Respirology [4] | 2014 | Non-invasive positive pressure ventilation | Standard oxygen therapy | In ICU | RoB 1.0 | 5 | RR : 0.69  [0.45; 1.07] | 0 |
| Santa Cruz et al.  Cochrane Database [5] | 2021 | High PEEP level | Low PEEP level | In hospital | RoB 1.0 | 7 | RR : 0.97  [0.90; 1.04] | 15 |
| Savoie-White et al.  Heart & Lung [6] | 2023 | Neuromuscular blockage | Standard of Care | Overall | RoB 2.0 | 6 | RR : 0.79  [0.62; 0.99] | 42 |
| Uhlig et al.  Critical Care [7] | 2014 | Albumin | Crystalloide | 28/31d | RoB 1.0 | 3 | RR : 0.89  [0.63; 1.28] | 0 |
| Walkey et al.  Annals of the American Thoracic society [8] | 2017 | Low tidal volume | Conventional tidal volume strategy | 28/31d | RoB 1.0 | 7 | RR : 0.87  [0.70; 1.08] | 46 |
| Zhong et al.  Ann Transl Med [9] | 2020 | Airway pressure release ventilation | Low tidal volume ventilation | In hospital | RoB 1.0 | 7 | OR : 0.57  [0.37; 0.88] | 0 |
| Cardiogenic shock | | | | | | | | |
| Uhlig et al.  Cochrane Database [10] | 2020 | Levosimendan | Dobutamine | 28/31d | Rob 2.0 | 4 | RR : 0.60  [0.36; 1.03] | 46 |
| Ouweneel et al.  J Am Coll Cardiol [11] | 2017 | IABP | Impella | 28/31d | Not reported | 3 | RR : 0.99  [0.62; 1.58] | NR |
| Altayyar et al.  Pol Arch Med Wewn [12] | 2015 | IABP | Placebo | 28/31d | RoB 1.0 | 4 | RR : 0.94  [0.79; 1.13] | 0 |
| Fernando et al.  Can J Anesth [13] | 2022 | Levosimendan | Placebo | Other | Rob 2.0 | 3 | RR : 0.53  [0.33; 0.87] | 0 |
| OHCA | | | | | | | | |
| Abuelazm et al.  Journal of Critical Care [14] | 2023 | Low MAP target | High MAP target | Overall | RoB 2.0 | 4 | RR : 1.07  [0.91; 1.27] | 0 |
| Al Lawati et al.  Critical Care Explorations [15] | 2023 | Early coronary angiography | Delayed coronary angiography | Overall | RoB 2.0 | 6 | RR : 1.04  [0.94; 1.15] | 0 |
| Faddy et al.  Cochrane Database [16] | 2016 | Biphasic waveforms defibrillation | Monophasic waveforms defibrillation | In hospital | RoB 1.0 | 4 | RR : 1.05  [0.78; 1.42] | 0 |
| Xu et al.  Journal of Critical Care [17] | 2024 | Low oxygen target | High oxygen target | Other | RoB 1.0 | 7 | RR : 0.98  [0.86; 1.11] | 28 |
| Sepsis or Septic shock | | | | | | | | |
| Liang et al.  Frontiers in Immunology [18] | 2021 | Corticosteroids | Placebo | 28/31d | RoB 2.0 | 40 | RR : 0.94  [0.87; 1.02] | 24 |
| Liu et al  Journal of Critical Care [19] | 2016 | Ulinastatin combined with thymosin alpha1 | Placebo | 28/31d | Jadad Scale | 8 | RR : 0.64  [0.54; 0.75] | 0 |
| Sivapalan et al.  Chest [20] | 2023 | High fluid volume | Low fluid volume | Overall | RoB 2.0 | 13 | RR : 0.98  [0.89; 1.08] | 0 |
| Sjövall et al.  Journal of Infection [21] | 2017 | Combination antibiotic | Monotherapy | Overall | RoB 1.0 | 10 | RR : 1.11  [0.95; 1.29] | 0 |
| Wang et al.  J Int Med Res. [22] | 2020 | Omega-3 fatty acid | Standard of Care | Overall | RoB 1.0 | 20 | RR : 0.82  [0.69; 0.97] | 0 |
| Wen et al.  Int J Clin Pract. [23] | 2023 | Vitamin C | Standard of Care | Overall | Jadad Scale | 22 | RR : 0.86  [0.74; 1.01] | 51 |
| Zhang et al.  Medicine [24] | 2022 | Esmolol | Standard of Care | 28/31d | RoB 1.0 | 5 | RR : 0.68  [0.52; 0.88] | 45 |
| Zhang et al.  Ann Intensive Care [25] | 2022 | Dexmedetomidine | Placebo | Overall | RoB 2.0 | 11 | RR : 0.83  [0.69; 0.99] | 1 |
| Zhang et al.  Plos One [26] | 2015 | Antipyretic therapy | Placebo | Overall | RoB 1.0 | 6 | OR : 1.02  [0.50; 2.05] | 71,7 |
| Meng et al.  Diabetes & Metabolic Syndrome: Clinical Research & Reviews [27] | 2024 | Intensive glucose control | Liberal glucose control | Overall | Rob 2.0 | 14 | RR : 1.06  [0.98; 1.16] | 0 |
| Zengzheng et al.  European Journal of Emergency Medicine [28] | 2024 | Levosimendan | Dobutamine or placebo | 28/31d | RoB 2.0 | 11 | OR : 0.93  [0.72; 1.20] | 0 |
| Pengyue et al.  Emergency and Critical Care Medicine [29] | 2024 | Clarythromycine | Placebo | 28/31d | RoB 1.0 | 3 | RR : 1.09  [0.87; 1.36] | 0 |
| Fujii et al.  Intensive Care Med [30] | 2018 | Polymyxin B-immobilized hemoperfusion | Standard of Care | 28/31d | RoB 1.0 | 5 | RR : 1.03  [0.78; 1.36] | 25 |
| Abdul-Aziz et al.  JAMA [31] | 2024 | Prolonged infusion of B-lactam | Intermittent infusion of B-lactam | 90d | Rob 2.0 | 17 | RR : 0.86  [0.72; 0.98] | 21,5 |
| Dari et al.  Cureus [32] | 2024 | High MAP target | Low MAP target | Overall | RoB 2.0 | 4 | RR : 0.94 [0.87; 1.01] | 0 |
| Thomas et al.  Minerva Anesthesiologica [33] | 2015 | Statin | Placebo | 28/31d | Jadad Scale | 4 | RR : 0.93  [0.72; 1.20] | 52 |
| Vasu et al.  Journal of Intensive Care Medicine [34] | 2012 | Norepinephrine | Dopamine | 28/31d | RoB 1.0 | 2 | RR : 0.91  [0.83; 0.99] | 0 |
| Li et al.  Medicine [35] | 2019 | Selenium | Placebo | 28/31d | RoB 2.0 | 12 | RR : 0.94  [0.82; 1.06] | 0 |
| Sangla et al.  Frontiers in Medicine [36] | 2023 | Thiamine | Placebo | Overall | RoB 1.0 | 5 | RR : 0.87  [0.65; 1.16] | 28 |
| Yao et al.  Front Pharmacol. [37] | 2020 | Vasopressine or Its Analogs | Standard of Care | 28/31d | Jadad Scale | 23 | RR : 0.94  [0.87; 1.01] | 0 |
| Fernando et al.  Can J Anesth [38] | 2024 | Methylene Blue | Placebo | Other | Rob 2.0 | 5 | RR : 0.66  [0.47; 0.94] | 0 |
| Patel et al.  Intensive Care Medicine [39] | 2013 | 6% tetrastarch | Any non-HES fluid | Overall | RoB 1.0 | 6 | RR : 1.13  [1.02; 1.25] | 0 |
| Lai et al.  Minerva Anestesiologica [40] | 2013 | Activated Drotrecogin Alfa | Saline | 28/31d | Not reported | 5 | RR : 0.97  [0.82; 1.14] | 59 |
| Trauma | | | | | | | | |
| Lyons et al.  Journal of the Royal Army Med Corps [41] | 2018 | Magnesium sulfate | Placebo | Overall | Jadad Scale | 6 | RR : 0.84  [0.54; 1.33] | 72 |
| Miyoshi et al.  Journal of Intensive Care [42] | 2020 | Hypertonic saline | Mannitol | Overall | RoB 1.0 | 3 | RR : 0.82  [0.49; 1.37] | 0 |

MA: Meta-analysis. RoB : Risk of bias. ARDS: Acute respiratory distress syndrome. OHCA: Out of Hospital Cardiac Arrest. ICU: Intensive care unit. PEEP: Positive end-expiratory pressure. IABP: Intra-aortic balloon pump. MAP: Mean arterial pressure. HES: Hydroxyethyl starch. RR: Risk ratio. OR: Odds ratio.

**S.Table 3.** Details of the Consent Process: from whom the consent was obtained and if it was written or orally obtained in RCTs with and without an RWPC Procedure

| Details of Consent process, n (%) | RCT with RWPC  N=59 | RCT without RWPC  N=264 |
| --- | --- | --- |
| From whom consent was obtained |  |  |
| SDMs or Patient | 46 (78) | 189 (72) |
| Patient only | 6 (10) | 26 (10) |
| SDMs only | 3 (5) | 49 (18) |
| Omitted | 4 (7) | - |
| How the consent was obtained |  |  |
| Written | 32 (54) | 139 (53) |
| Oral | 2 (3) | 1 (1) |
| Not reported | 26 (44) | 124 (47) |

RCT: Randomized controlled trial. RWPC: Research without prior consent. SDM: Surrogate decision maker

**S.Figure 2.** Part of RWPC procedure in RCTs in each decade since 1980

**
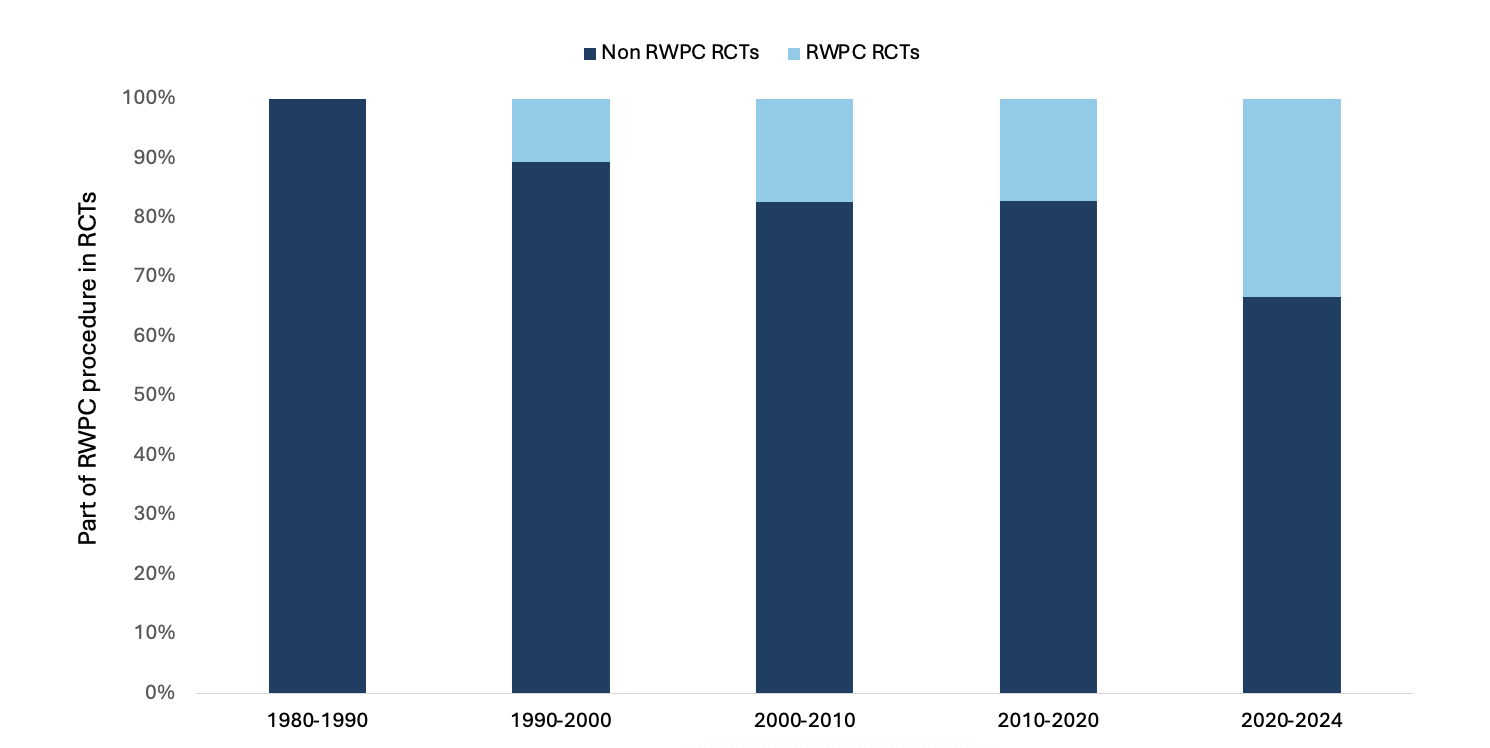
**

RWPC: Research without prior consent. RCT: Randomized controlled trial

**S. Figure 3.** Contour-enhanced funnel plot of meta-analysis by Anand et al. Zones of statistical significance are annotated directly within the plot (*** for p ≤ 0.01, ** for 0.01 < p ≤ 0.05, and * for 0.05 < p ≤ 0.10). Trials involving research without prior consent (RWPC) are shown in light blue; non-RWPC trials in dark blue.


**S. Figure 4.** Contour-enhanced funnel plot of meta-analysis by Cao et al. Zones of statistical significance are annotated directly within the plot (*** for p ≤ 0.01, ** for 0.01 < p ≤ 0.05, and * for 0.05 < p ≤ 0.10). Trials involving research without prior consent (RWPC) are shown in light blue; non-RWPC trials in dark blue.

**S. Figure 5.** Contour-enhanced funnel plot of meta-analysis by Anand et al. Zones of statistical significance are annotated directly within the plot (*** for p ≤ 0.01, ** for 0.01 < p ≤ 0.05, and * for 0.05 < p ≤ 0.10). Trials involving research without prior consent (RWPC) are shown in light blue; non-RWPC trials in dark blue.

**S. Figure 6.** Contour-enhanced funnel plot of meta-analysis by Liang et al. Zones of statistical significance are annotated directly within the plot (*** for p ≤ 0.01, ** for 0.01 < p ≤ 0.05, and * for 0.05 < p ≤ 0.10). Trials involving research without prior consent (RWPC) are shown in light blue; non-RWPC trials in dark blue.

**S. Figure 7.** Contour-enhanced funnel plot of meta-analysis by Sivapalan et al. Zones of statistical significance are annotated directly within the plot (*** for p ≤ 0.01, ** for 0.01 < p ≤ 0.05, and * for 0.05 < p ≤ 0.10). Trials involving research without prior consent (RWPC) are shown in light blue; non-RWPC trials in dark blue.

**S. Figure 8.** Contour-enhanced funnel plot of meta-analysis by Wen et al. Zones of statistical significance are annotated directly within the plot (*** for p ≤ 0.01, ** for 0.01 < p ≤ 0.05, and * for 0.05 < p ≤ 0.10). Trials involving research without prior consent (RWPC) are shown in light blue; non-RWPC trials in dark blue.

**S. Figure 9.** Contour-enhanced funnel plot of meta-analysis by Yao et al. Zones of statistical significance are annotated directly within the plot (*** for p ≤ 0.01, ** for 0.01 < p ≤ 0.05, and * for 0.05 < p ≤ 0.10). Trials involving research without prior consent (RWPC) are shown in light blue; non-RWPC trials in dark blue.


**S. Figure 10.** Contour-enhanced funnel plot of meta-analysis by Meng et al. Zones of statistical significance are annotated directly within the plot (*** for p ≤ 0.01, ** for 0.01 < p ≤ 0.05, and * for 0.05 < p ≤ 0.10). Trials involving research without prior consent (RWPC) are shown in light blue; non-RWPC trials in dark blue.

**S. Figure 11.** Contour-enhanced funnel plot of meta-analysis by Abdul-Aziz et al. Zones of statistical significance are annotated directly within the plot (*** for p ≤ 0.01, ** for 0.01 < p ≤ 0.05, and * for 0.05 < p ≤ 0.10). Trials involving research without prior consent (RWPC) are shown in light blue; non-RWPC trials in dark blue.

**S. Figure 12.** Subgroup analysis of the difference in treatment effect estimate on mortality between RCTs using a RWPC procedure RCTs not using a RWPC procedure according to the type of intervention.

RWPC: Research without prior consent. RCT: Randomized controlled trial. ROR: Ratio of odds ratios. CI: Confidence Interval.

**S. Figure 13.** Subgroup analysis of the difference in treatment effect estimate on mortality between RCTs using a RWPC procedure RCTs not using a RWPC procedure according to the medical condition.

RWPC: Research without prior consent. RCT: Randomized controlled trial. ROR: Ratio of odds ratios. CI: Confidence Interval.

**S. Figure 14.** Difference in treatment effect estimate on mortality between RCTs using a RWPC procedure RCTs not using a RWPC procedure: sensitivity analysis adjusted for overall risk of bias, date of publication, multicenter design and sample size


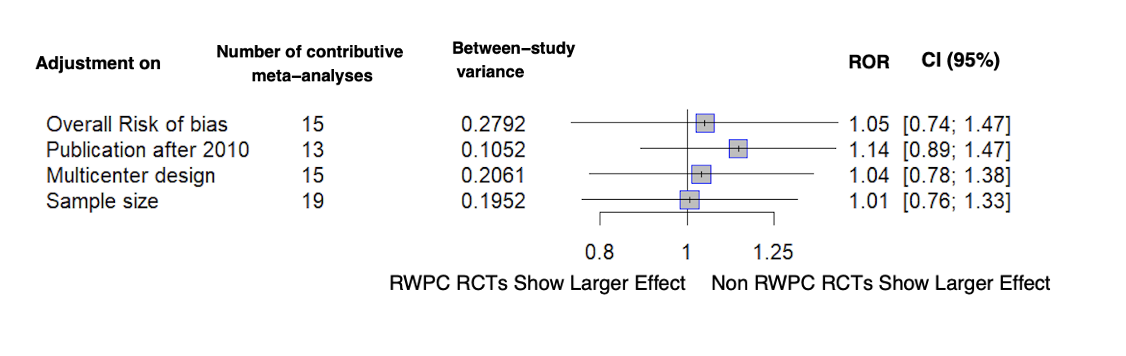


RWPC: Research without prior consent. RCT: Randomized controlled trial. ROR: Ratio of odds ratios. CI: Confidence Interval.

**S.Table 4**. Characteristics of included RCTs by single-center versus multicenter design

|  |  | Single-center Trials N= 163 | Multicenter Trials N= 160 | p |
| --- | --- | --- | --- | --- |
| RWPC, n (%) | | 12 (8) | 47 (29) | <0.001 |
| Medical condition, n (%) | |  |  | <0.001 |
|  | - Sepsis | 68 (42) | 68 (42) |  |
|  | - Septic shock | 58 (36) | 29 (18) |  |
|  | - ARDS | 19 (12) | 39 (24) |  |
|  | - Cardiac arrest | 7 (4) | 14 (9) |  |
|  | - Cardiogenic shock | 5 (3) | 8 (5) |  |
|  | - Trauma | 6 (4) | 2 (1) |  |
| Intervention type, n (%) | |  |  | 0.014 |
|  | - Pharmacological | 117 (72) | 93 (58) |  |
|  | - Non-pharmacological | 46 (28) | 67 (42) |  |
| Control group, n (%) | |  |  | 0.002 |
|  | - Placebo | 60 (37) | 50 (31) |  |
|  | - Standard of care | 31 (19) | 15 (9) |  |
|  | - No intervention | 1 (1) | 9 (6) |  |
|  | - Other intervention | 71 (44) | 86 (54) |  |

RWPC: Research without prior consent. ARDS: Acute respiratory distress syndrome.

**References**

1. Anand R, McAuley DF, Blackwood B, Yap C, ONeill B, Connolly B, et al. Mucoactive agents for acute respiratory failure in the critically ill: a systematic review and meta-analysis. Thorax. 2020;75:623–31.

2. Cao Z, Yang Z, Liang Z, Cen Q, Zhang Z, Liang H, et al. Prone versus Supine Position Ventilation in Adult Patients with Acute Respiratory Distress Syndrome: A Meta-Analysis of Randomized Controlled Trials. Emerg Med Int. 2020;2020:4973878.

3. Feng Y. Efficacy of statin therapy in patients with acute respiratory distress syndrome/acute lung injury: a systematic review and meta-analysis. Eur Rev Med Pharmacol Sci. 2018;22:3190–8.

4. Luo J, Wang M, Zhu H, Liang B, Liu D, Peng X, et al. Can non-invasive positive pressure ventilation prevent endotracheal intubation in acute lung injury/acute respiratory distress syndrome? A meta-analysis. Respirology. 2014;19:1149–57.

5. Santa Cruz R, Villarejo F, Irrazabal C, Ciapponi A. High versus low positive end-expiratory pressure (PEEP) levels for mechanically ventilated adult patients with acute lung injury and acute respiratory distress syndrome. Cochrane Database Syst Rev. 2021;3:CD009098.

6. Savoie-White FH, Tremblay L, Menier CA, Duval C, Bergeron F, Tadrous M, et al. The use of early neuromuscular blockage in acute respiratory distress syndrome: A systematic review and meta-analyses of randomized clinical trials. Heart Lung. 2023;57:186–97.

7. Uhlig C, Silva PL, Deckert S, Schmitt J, de Abreu MG. Albumin versus crystalloid solutions in patients with the acute respiratory distress syndrome: a systematic review and meta-analysis. Crit Care. 2014;18:R10.

8. Walkey AJ, Goligher EC, Del Sorbo L, Hodgson CL, Adhikari NKJ, Wunsch H, et al. Low Tidal Volume versus Non-Volume-Limited Strategies for Patients with Acute Respiratory Distress Syndrome. A Systematic Review and Meta-Analysis. Ann Am Thorac Soc. 2017;14:S271–9.

9. Zhong X, Wu Q, Yang H, Dong W, Wang B, Zhang Z, et al. Airway pressure release ventilation versus low tidal volume ventilation for patients with acute respiratory distress syndrome/acute lung injury: a meta-analysis of randomized clinical trials. Ann Transl Med. 2020;8:1641.

10. Uhlig K, Efremov L, Tongers J, Frantz S, Mikolajczyk R, Sedding D, et al. Inotropic agents and vasodilator strategies for the treatment of cardiogenic shock or low cardiac output syndrome. Cochrane Database Syst Rev. 2020;11:CD009669.

11. Ouweneel DM, Eriksen E, Seyfarth M, Henriques JPS. Percutaneous Mechanical Circulatory Support Versus Intra-Aortic Balloon Pump for Treating Cardiogenic Shock: Meta-Analysis. Journal of the American College of Cardiology. 2017;69:358–60.

12. Altayyar S, Al-Omari A, Alqahtani AM, Rochwerg B, Alnasser S, Alqahtani Z, et al. Intraaortic balloon pump in patients with cardiogenic shock complicating myocardial infarction: a systematic review and meta-analysis of randomized trials. Pol Arch Med Wewn. 2015;125:181–90.

13. Fernando SM, Mathew R, Sadeghirad B, Brodie D, Belley-Côté EP, Thiele H, et al. Inotropes, vasopressors, and mechanical circulatory support for treatment of cardiogenic shock complicating myocardial infarction: a systematic review and network meta-analysis. Can J Anaesth. 2022;69:1537–53.

14. Abuelazm M, Ali S, Mahmoud A, Mechi A, Kadhim H, Katamesh BE, et al. High versus low mean arterial pressure targets after out-of-hospital cardiac arrest: A systematic review and meta-analysis of randomized controlled trials. J Crit Care. 2023;78:154365.

15. Al Lawati K, Forestell B, Binbraik Y, Sharif S, Ainsworth C, Mathew R, et al. Early Versus Delayed Coronary Angiography After Out-of-Hospital Cardiac Arrest Without ST-Segment Elevation-A Systematic Review and Meta-Analysis of Randomized Controlled Trials. Crit Care Explor. 2023;5:e0874.

16. Faddy SC, Jennings PA. Biphasic versus monophasic waveforms for transthoracic defibrillation in out-of-hospital cardiac arrest. Cochrane Database Syst Rev. 2016;2:CD006762.

17. Xu Y, Peng F, Wang S, Yu H. Lower versus higher oxygen targets after resuscitation from out-of-hospital cardiac arrest: A systematic review and meta-analysis of randomized controlled trials. J Crit Care. 2024;79:154448.

18. Liang H, Song H, Zhai R, Song G, Li H, Ding X, et al. Corticosteroids for Treating Sepsis in Adult Patients: A Systematic Review and Meta-Analysis. Front Immunol. 2021;12:709155.

19. Liu D, Yu Z, Yin J, Chen Y, Zhang H, Fan X, et al. Effect of ulinastatin combined with thymosin alpha1 on sepsis: A systematic review and meta-analysis of Chinese and Indian patients. J Crit Care. 2017;39:285–7.

20. Sivapalan P, Ellekjaer KL, Jessen MK, Meyhoff TS, Cronhjort M, Hjortrup PB, et al. Lower vs Higher Fluid Volumes in Adult Patients With Sepsis: An Updated Systematic Review With Meta-Analysis and Trial Sequential Analysis. Chest. 2023;164:892–912.

21. Sjövall F, Perner A, Hylander Møller M. Empirical mono- versus combination antibiotic therapy in adult intensive care patients with severe sepsis - A systematic review with meta-analysis and trial sequential analysis. J Infect. 2017;74:331–44.

22. Wang C, Han D, Feng X, Wu J. Omega-3 fatty acid supplementation is associated with favorable outcomes in patients with sepsis: an updated meta-analysis. J Int Med Res. 2020;48:300060520953684.

23. Wen C, Li Y, Hu Q, Liu H, Xu X, Lü M. IV Vitamin C in Sepsis: A Latest Systematic Review and Meta-Analysis. Int J Clin Pract. 2023;2023:6733465.

24. Zhang J, Chen C, Liu Y, Yang Y, Yang X, Yang J. Benefits of esmolol in adults with sepsis and septic shock: An updated meta-analysis of randomized controlled trials. Medicine (Baltimore). 2022;101:e29820.

25. Zhang T, Mei Q, Dai S, Liu Y, Zhu H. Use of dexmedetomidine in patients with sepsis: a systematic review and meta-analysis of randomized-controlled trials. Ann Intensive Care. 2022;12:81.

26. Zhang Z. Antipyretic therapy in critically ill patients with established sepsis: a trial sequential analysis. PLoS One. 2015;10:e0117279.

27. Meng J, Li X, Xiao Y, Tang H, Liu P, Wu Y, et al. Intensive or liberal glucose control in intensive care units for septic patients? A meta-analysis of randomized controlled trials. Diabetes & Metabolic Syndrome: Clinical Research & Reviews. 2024;18:103045.

28. Ge Z, Gao Y, Lu X, Yu S, Qin M, Gong C, et al. The association between levosimendan and mortality in patients with sepsis or septic shock: a systematic review and meta-analysis. European Journal of Emergency Medicine. 2024;31:90.

29. Zhao P, Yao R, Yang J, Wen W, Yao Y, Du X. Efficacy and safety of clarithromycin for patients with... : Emergency and Critical Care Medicine. [cited 2025 Jun 6]; Available from: https://journals.lww.com/eccm/fulltext/2024/06000/efficacy_and_safety_of_clarithromycin_for_patients.7.aspx

30. Fujii T, Salanti G, Belletti A, Bellomo R, Carr A, Furukawa TA, et al. Effect of adjunctive vitamin C, glucocorticoids, and vitamin B1 on longer-term mortality in adults with sepsis or septic shock: a systematic review and a component network meta-analysis. Intensive Care Med. 2022;48:16–24.

31. Abdul-Aziz MH, Hammond NE, Brett SJ, Cotta MO, De Waele JJ, Devaux A, et al. Prolonged vs Intermittent Infusions of β-Lactam Antibiotics in Adults With Sepsis or Septic Shock: A Systematic Review and Meta-Analysis. JAMA. 2024;332:638–48.

32. Dari MA, Fayaz A, Sharif S, Hernandez Galaviz S, Hernandez Galaviz E, Bataineh SM, et al. Comparison of High-Normal Versus Low-Normal Mean Arterial Pressure at Target on Outcomes in Sepsis or Shock Patients: A Meta-Analysis of Randomized Control Trials. Cureus. 2024;16:e52258.

33. Thomas G, Hraiech S, Loundou A, Truwit J, Kruger P, Mcauley DF, et al. Statin therapy in critically-ill patients with severe sepsis: a review and meta-analysis of randomized clinical trials. Minerva Anestesiol. 2015;81:921–30.

34. Vasu TS, Cavallazzi R, Hirani A, Kaplan G, Leiby B, Marik PE. Norepinephrine or dopamine for septic shock: systematic review of randomized clinical trials. J Intensive Care Med. 2012;27:172–8.

35. Li S, Tang T, Guo P, Zou Q, Ao X, Hu L, et al. A meta-analysis of randomized controlled trials: Efficacy of selenium treatment for sepsis. Medicine (Baltimore). 2019;98:e14733.

36. Sangla F, Verissimo T, Faivre A, Glauser T, Cheah SK, Assouline B, et al. Thiamine as a metabolic resuscitator in septic shock: a meta-analysis of randomized controlled trials with trial sequential analysis. Front Med (Lausanne). 2023;10:1223862.

37. Yao R-Q, Xia D-M, Wang L-X, Wu G-S, Zhu Y-B, Zhao H-Q, et al. Clinical Efficiency of Vasopressin or Its Analogs in Comparison With Catecholamines Alone on Patients With Septic Shock: A Systematic Review and Meta-Analysis. Front Pharmacol. 2020;11:563.

38. Fernando SM, Tran A, Soliman K, Flynn B, Oommen T, Wenzhe L, et al. Methylene Blue in Septic Shock: A Systematic Review and Meta-Analysis. Critical Care Explorations. 2024;6:e1110.

39. Patel A, Waheed U, Brett SJ. Randomised trials of 6% tetrastarch (hydroxyethyl starch 130/0.4 or 0.42) for severe sepsis reporting mortality: systematic review and meta-analysis. Intensive Care Med. 2013;39:811–22.

40. Lai PS, Matteau A, Iddriss A, Hawes JCL, Ranieri V, Thompson BT. An updated meta-analysis to understand the variable efficacy of drotrecogin alfa (activated) in severe sepsis and septic shock. Minerva Anestesiol. 2013;79:33–43.

41. Lyons MWH, Blackshaw WJ. Does magnesium sulfate have a role in the management of severe traumatic brain injury in civilian and military populations? A systematic review and meta-analysis. J R Army Med Corps. 2018;164:442–9.

42. Miyoshi Y, Kondo Y, Suzuki H, Fukuda T, Yasuda H, Yokobori S. Effects of hypertonic saline versus mannitol in patients with traumatic brain injury in prehospital, emergency department, and intensive care unit settings: a systematic review and meta-analysis. J Intensive Care. 2020;8:61.
