# Supplementary material for: Research without prior consent procedure and intervention effect on mortality in critical care: a meta-epidemiological study of randomized controlled trials
Source: Crit Care. 2025 Jul 24;29:323. doi: 10.1186/s13054-025-05480-x (PMC12291516; doi:10.1186/s13054-025-05480-x)
Supplement: Supplementary file 2 — Supplementary Material 2 [file 13054_2025_5480_MOESM2_ESM.docx]

Data Extraction Form for **Meta-analyses**: Association between emergency consent procedure and treatment effect on mortality in randomized controlled trials in critical care: protocol for a meta-epidemiological study

**Reviewer:** GH [ ] JL [ ]

**Number meta-analysis:** |__||__||__| **Date of publication (year):** |__||__||__||__|

**Title:** ______________________________________________________________

**First author:** ______________________________________________________________

**Journal:** ______________________________________________________________

**Funding sources:** Public [ ] Private [ ] Public and private [ ]

No specific funding? [ ]

**Number of Trials in the meta-analysis of mortality:** |__||__||__|

Number of participants in the meta-analysis of mortality: |__||__||__||__||__|

**Medical Condition:**

**Septic shock**  [ ]

**Cardiogenic shock**  [ ]

**Hemorrhagic shock**  [ ]

**Sepsis**  [ ]

**ARDS**  [ ]

**Trauma**  [ ]

**Cardiac arrest**  [ ]

**Other**  [ ]

**Control treatment:**

Placebo [ ] No treatment [ ] Other: __________________________

Intervention details:

Pharmacologic [ ] Non-pharmacologic [ ]

Name: _____________________________________________________

**Experimental treatment 1:**

Pharmacologic [ ] Non-pharmacologic [ ]

Name: _____________________________________________________

**Experimental treatment 2:**

Pharmacologic [ ] Non-pharmacologic [ ]

Name: _____________________________________________________

**Mortality:**

Primary outcome [ ] Secondary outcome [ ]

Time point of assessment:

- ICU [ ]
- 28-31 days [ ]
- 90 days [ ]
- In hospital [ ]
- Overall [ ]
- Other [ ]

**Tool used to assess risk of bias:**

- Cochrane Risk of bias Tool (RoB.1) [ ]
- RoB.2[ ]
- Jadad scale[ ]
- Other[ ]

**Method for pooling data**

- Fixed effect model [ ]
- Random effect model [ ]
- Inverse variance weighting[ ]
- Metaregression[ ]
- Other[ ]

**Results of the meta-analysis for mortality**

- CI [ ]
- I2 [ ]

Data Extraction Form for an **individual RCT**: Association between emergency consent procedure and treatment effect on mortality in randomized controlled trials in critical care: protocol for a meta-epidemiological study

**Reviewer:** GH [ ] JL [ ]

**Number RCT:** |__||__||__| **Date of publication (year):** |__||__||__||__|

**Title:** ______________________________________________________________

**First author:** ______________________________________________________________

**Journal:** ______________________________________________________________

**Funding sources:** Public [ ] Private [ ] Public and private [ ]

No specific funding? [ ]

**Monocenter study:** Yes, monocenter [ ] No, multicenter [ ] Not reported [ ]

- If multicenter study, how many centers: |__||__||__||__|

**Countries:** ______________________________________________________________

**Sample size:**  |__||__||__|

**Ethical Considerations:**

RWPC with an emergency consent procedure/deferred consent? Yes [ ] No [ ]

**Emergency consent procedure used:**

Consent obtained once the patient’s consciousness is regained? Yes [ ] No [ ]

Consent obtained from relative? Yes [ ] No [ ]

In patient death occurred, how is data used:

- Data used: Yes [ ] No [ ]
- Relative consent for data usage: Yes [ ] No [ ]

**Emergency consent procedure absent:**

Method of obtaining consent: Written [ ] Oral [ ]

Data acquired directly? Yes, from patient [ ] Yes, from relative [ ]

Not obtained [ ]

**Performance of analysis:**

Intention to treat [ ] Per-protocol [ ]

Number of consent withdrawal in ITT analysis? Yes [ ] No [ ]

Estimated consent withdrawal in sample size calculation? Yes [ ] No [ ]

**Patient characteristics:**

Sample size: |__||__||__||__||__||__|

| Inclusion criteria |  |
| --- | --- |
| Exclusion criteria |  |

Consent withdrawal per group:

- Group __________________**:** |__||__||__||__|
- Group ___________________: |__||__||__||__|
- Group ___________________: |__||__||__||__|

**Time intervals:**

Between trial eligibility and randomization: ___________________

Between ICU admission and randomization: ___________________

**Primary outcome:** ________________________________________________________________________

Definition: _______________________________________________

Time of assessment: |__||__| days

**Mortality:**

Time point of assessment:

- ICU [ ]
- 28-31 days [ ]
- 90 days [ ]
- In hospital [ ]
- Overall [ ]
- Other [ ]

**Group control**

Number of analyzed patients:

Number of event (mortality):

**Group Experimental**

Number of analyzed patients:

Number of event (mortality):
